# Long non-coding RNA GEHT1 promoted the proliferation of ovarian cancer cells via modulating the protein stability of HIF1α

**DOI:** 10.1042/BSR20181650

**Published:** 2019-05-07

**Authors:** Dan Liu, Hao Li

**Affiliations:** Department of Obstetrics & Gynecology, The People’s Hospital of Hanchuan city, Hubei, China, 431600

**Keywords:** GHET1, HIF1a, Ovarian cancer, VHL

## Abstract

Cancer cells preferentially metabolize glucose via the aerobic glycolysis pathway, which is also named as Warburg effect. Increasing evidence has suggested that suppression of glycolysis inhibits the progression of cancers. In the present study, we found that the long non-coding RNA gastric carcinoma high expressed transcript 1 (GHET1) was overexpressed in ovarian cancer tissues and cell lines. Up-regulation of GHET1 was positively correlated with the tumor size and metastasis of the ovarian cancer patients. Overexpression of GEHT1 significantly promoted the proliferation and colony formation of ovarian cancer cells. Mechanistically, the candidate binding partners of GHET1 were explored by pull-down and mass spectrum. Of note, GHET1 was found to interact with the E3 ubiquitin ligase von Hippel-Lindau (VHL), which consequently blocked VHL-mediated degradation of hypoxia-inducible factor-1α (HIF1α) and enhanced the protein level of HIF1α in ovarian cancer cells. The up-regulated HIF1α promoted the glucose uptake and lactate generation of ovarian cancer cells. Collectively, our results suggested the oncogenic function of GHET1 via up-regulating the glycolysis in ovarian cancer and can be considered as a promising anti-cancer target.

## Introduction

Ovarian cancer has been the most lethal gynecological malignancy worldwide. Even remarkable progress has been made in understanding the initiation and progression of ovarian cancer, the incidence and mortality rates of the ovarian cancer patients still remains very high [1–3]. Therefore, exploring the pathophysiological mechanisms that contribute to the development of ovarian cancer is quite vital for developing novel effective therapies.

Long non-coding RNAs (lncRNAs) are non-protein coding transcripts, which are longer than 200 nucleotides and play important roles in regulating cell proliferation, differentiation and apoptosis [4–8]. Aberrant expression of lncRNAs has been observed in human diseases, especially cancers [5,9]. LncRNAs modulate the progression of cancers via oncogenic or tumor suppressive roles. Due to the critical function of lncRNAs in regulating the gene expression, investigating novel lncRNAs that might be involved in the initiation and development of ovarian cancer is necessary. Recent study identified a novel long noncoding RNA, named gastric carcinoma high expressed transcript 1 (GHET1), which was overexpressed in gastric cancer tissues and correlated with the worse prognosis of gastric cancer patients [10–12]. Further mechanism study uncovered that GHET1 promoted the proliferation of gastric cancer cells via modulating the stability and expression of the oncogene c-Myc [12]. Additionally, GHET1 was also found overexpressed in bladder cancer cell lines and tissues [13]. Highly expressed GHET1 enhanced the proliferation and invasion via regulating the epithelial–mesenchymal transition (EMT) process. These finding suggested the potential involvement of GHET1 in tumor progression and might be considered as a novel target in designing therapeutic strategy of cancers. However, the expression and function of GHET1 in ovarian cancer have not been demonstrated.

Glucose metabolism is an essential process to maintain the cell growth and individual development. Unlike the normal cells, tumor cells metabolize glucose through the aerobic glycolysis instead of the mitochondrial respiration, which is also known as Warburg effect [14]. In addition to providing cellular energy, the glycolysis provides metabolic intermediates for the macromolecular biosynthesis, which facilitates the rapid growth of cancer cells [15]. Therefore, targeting the glycolysis is a promising therapeutic intervention of cancers. Oncogenes or tumor suppressors have been found to modulate the glucose metabolism of cancer cells via regulating the expression of enzymes involved in the aerobic glycolysis [14]. Among the essential components of glycolysis, the hypoxia-inducible factor-1α (HIF1α) is a key regulator of the hypoxic response [16]. Under normal condition, HIF1α is hydroxylated by the proline hydroxylase domain (PHD) protein, which results in the interaction of HIF1α with the E3 ubiquitination ligase von Hippel-Lindau (VHL) [17]. The binding between HIF1α and VHL leads to the proteasome dependent degradation of HIF1α. However, under hypoxic conditions, the activity of PHD is inhibited and HIF1α is stabilized to dimerize with HIF-1β to up-regulate the expression of enzymes in the glycolysis. These enzymes include the glucose transporters and the lactate dehydrogenase A (LDHA), which promote the Warburg effect [18,19]. Given the critical roles of HIF1α in modulating the growth of cancer cells, interfering the expression of HIF1α is an attractive strategy to inhibit the malignancy of cancers.

In the present study, we showed that GHET1 was highly expressed in ovarian cancer tissues. Overexpression of GHET1 promoted the proliferation of ovarian cancer cell. The molecular study uncovered that GHET1 interacted with VHL and blocked the binding between VHL and HIF1α, which led to the stabilization of HIF1α and up-regulation of glycolysis of ovarian cancer cells.

## Materials and methods

### Tissues and cell lines

The 50 epithelial specimens of normal ovary and 50 specimens of ovarian cancer tissues were obtained from the patients at the People’s Hospital of Hanchuan city during March 2010 to October 2013. None of the participants received therapies before the surgery. All the tissues were stored at liquid nitrogen until use. The study was approved by the ethics committee of The People’s Hospital of Hanchuan city in accordance with the World Medical Association Declaration of Helsinki, and that all subjects provided written informed consent.

The normal human ovarian surface epithelial cells normal epithelial ovarian epithelial cells (HOSEpiC) and ovarian cancer cell lines OVCAR3, SKOV3, 3AO and A2780 were purchased from the American Type Culture Collection (ATCC, Manassas, VA, U.S.A.). Cells were cultured with the DMEM medium containing 10% fetal bovine serum (FBS, Corning, NY, U.S.A.) and 1% streptomycin/penicillin at 37°C with 5% CO_2_.

### RNA extraction and RT-qPCR analysis

Total RNA was isolated from the tissues or cells with the Trizol reagent (Millipore, Billerica, MA, U.S.A.). The RNA concentration was determined with the NanoDrop-2000 (ThermoFisher Inc., CA, U.S.A.). Reverse transcription was performed with 1 μg of RNA using the PrimeScript RT master Mix (Takara, Shiga, Japan). The expression of GHET1 was evaluated by the real-time PCR assay with the SYBR Premix Ex Taq™ kit (Takara, Shiga, Japan) on the 7900 Fast Real-time PCR system (Applied Biosystem). The level of GAPDH was also detected as the endogenous control. The primers of GHET1 were as follows: forward, 5′-CCCCACAAATGAAGACACT and reverse, 5′-TTCCCAACACCCTATAAGAT; GAPDH, forward, 5′-GTCGGTGTGAACGGATTTG and reverse, 5′-AAGATGGTGATGGGCTTCC. The PCR reaction was set as 95°C for 10 min; 40 cycles at 95°C for 10 sec and 60°C for 1 min. The relative expression of GHET1 was calculated with the 2^−ΔΔ*C*^_q_ method.

### Dimethyl thiazolyl diphenyl tetrazolium assay

The 2000 cells transfected with plasmid expressing GHET1 or control lncRNA were seeded in the 96-well plate per well. After culturing for the indicated time, 20 μl of 5 mg/ml dimethyl thiazolyl diphenyl tetrazolium (MTT) reagent (Millipore, Billerica, MA, U.S.A.) was added into the wells and incubated at 37°C for 4 h. Afterward, 200 μl of dimethyl sultoxide was added into the cells and the absorbance of each well was measured at 550 nm. The experiment was performed in triplicate.

### Western blot

Cells were transfected with GHET1 or control vector for 48 h. After that, cells were lysed with the RIPA lysis buffer (Beyotime, Shanghai, China) and the protein concentration was determined with the BCA assay (Beyotime, Shanghai, China). The 20 μg of protein was separated by the SDS/PAGE and transferred onto the polyvinylidene fluoride membrane (Millipore, Billerica, MA, U.S.A.). After blocking with 5% non-fat milk, the membrane was incubated with the primary antibody overnight at 4°C. And then the membrane was incubated with the horseradish peroxidase labeled secondary antibody for 1 h at room temperature. The protein bands were visualized with the ECL detection kit (Beyotime, Shanghai, China). The antibodies including anti-HIF1α (#36169), anti-VHL (#68547), anti-vimentin (#5741), anti-N-cadherin (#13116) and anti-E-cadherin (#14472) were commercially purchased from Cell Signaling Technology (Danvers, MA, U.S.A.).

### Determination of the cell apoptosis

The apoptosis of cells expressing GHET1 or control lncRNA was determined with the Annexin V fluorescein-5-isothiocyanate (FITC) apoptosis detection kit (Invitrogen, ThermoFisher Inc., U.S.A.) according to the manufacturer’s instructions. After transfection for 48 h, cells were harvested and washed twice with pre-cold PBS. Cells were then resuspended in 1 × Annexin V binding buffer. The 100 μl of cell resuspension (∼10000 cells) was stained with FITC-Annexin V and propidium iodide for 15 min at room temperature. The percentage of cell apoptosis was analyzed with the flow cytometry.

### Colony formation assay

Both A780 and SKOV3 cells were transfected with lncRNA control vector or GHET1 and seeded in the 35 mm plate with 1000 cells per dish. Cells were cultured with DMEM medium containing 10% FBS. 2 weeks after transfection, colonies were washed twice with PBS and fixed with 4% paraformaldehyde for 10 min at RT. The colonies were then stained with 1% crystal violet at RT for 15 min. After washing with PBS, the colony formation was observed with the light microscopy and the number of the colonies was counted.

### LncRNA pull-down and mass spectrometry

The full-length antisense of GHET1 RNA was *in vitro* transcribed with the MEGAscript™ T7 Transcription kit (Thermo Fisher Scientific, Inc., Waltham, MA, U.S.A.) according to the manufacturer’s instructions. The antisense transcript was labeled with biotin using the Pierce™ RNA 3′ End Desthiobiotinylation kit (Thermo Fisher Scientific, Inc., Waltham, MA, U.S.A.). A2780 cells were harvested and lysed with the NP-40 lysis buffer. The whole cell lysis was incubated with the biotin labeled antisense of GHET1 for 4 h at 4°C. Streptavidin beads were added into the lysis and incubated for 1 h at 4°C. The beads were obtained by centrifugation for 5 min at 3000 rpm at 4°C. The potential binding proteins of GHET1 was analyzed with 15% SDS/PAGE. The protein signal was visualized using the Sliver Stain Plus™ kit (#1610449, Bio-Rad Laboratories, Inc., Hercules, CA, U.S.A.). The mass spectrometry was analyzed with the Matrix Assisted Laser Desorption Ionization Time of Flight Mass Spectrometry (CapitalBio Technology Biocompany, Beijing, China).

### Glucose uptake assay

Ovarian cancer cells were transfected with the corresponding lncRNA and seeded in the 96-well plate. The glucose consumption of the cells was determined with the Glucose Uptake Colorimetric Assay kit (BioVision, Milpitas, CA, U.S.A.) according to the manufacturer’s instructions. Briefly, both A780 and SKOV3 cells were starved with the Krebs-Ringer-Phosphate-HEPES buffer supplemented with 2% BSA for 30 min. And then 10 mM 2-DG was added into the medium and incubated for 20 min. Following this step, the medium was discarded and cells were washed with PBS for three-times at RT. After centrifugation at 2000 rpm for 5 min, cells were resuspended in 3 ml of 10 mM Tris-HCl buffer (pH 8.0) and disrupted with the microtip sonicator. Samples were heated at 80°C for 15 min and then centrifuged at 15000 ***g*** for 20 min at 4°C. The supernatant was collected into another tube and the glucose uptake of each group was determined according to the instructions. The experiment was performed in triplicated. The protein concentration was detected for the normalization between different groups.

### Measurement of the lactate production

Lactate production of the ovarian cells expressing corresponding lncRNA was determined with the Lactate Assay Kit (MAK064, Sigma–Aldrich, U.S.A.) according to the manufacturer’s instructions. After transfection for 48 h, cells were lysed with the indicated buffer for 15 min on ice. The samples were centrifuged at 13000 ***g*** for 10 min at 4°C and the supernatant was collected. The 50 μl of supernatant was added into the 96-well plate and mixed with equal volume of master reaction buffer for 30 min at RT. The standard lactate solution curved was obtained by diluting 0, 2, 4, 6, 8 and 10 μl of the lactate standard into the 96-well plate. The lactate generation was determined by measuring the absorbance of each well at 570 nm with the microplate reader (Bio-Rad, Laboratories, Inc., Hercules, CA, U.S.A.).

### Statistical analysis

The data were presented as mean ± S.D. The statistical analysis was performed using the Student’s *t* test or one-way ANOVA analysis with the SPSS 13.0 (SPSS, Inc., Chicago, IL, U.S.A.). The *P*<0.05 was considered as statistical significant.

## Results

### GHET1 was up-regulated in ovarian cancer tissues and was associated with the poor prognosis of the patients

**Figure 1:**
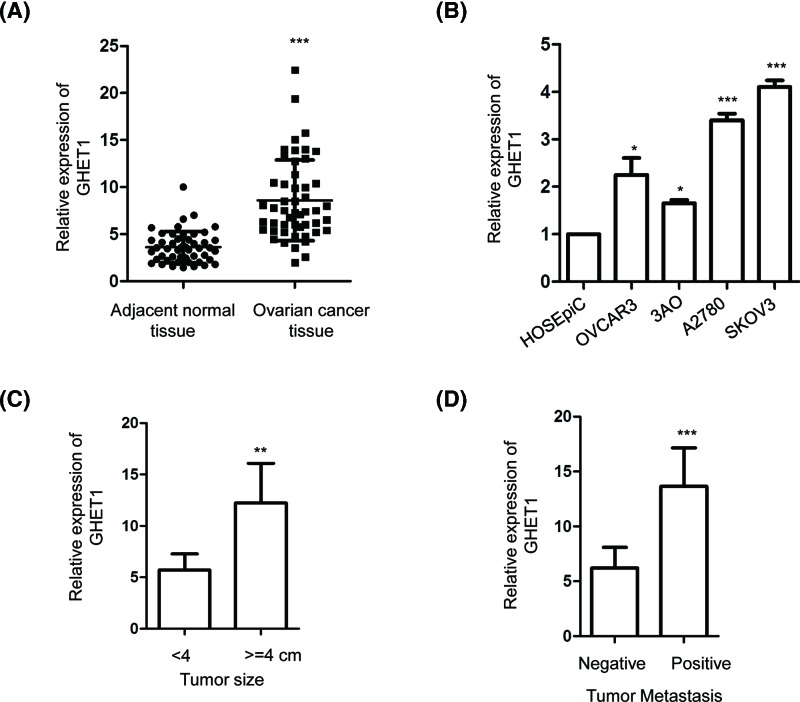
GHET1 was overexpressed in ovarian cancer tissues and cell lines (**A**) Expression of GHET1 in paired ovarian cancer tissues and adjacent normal tissues were detected by RT-qPCR. (**B**) The level of GHET1 in normal HOSEpiC cells and ovarian cancer cell lines OVCAR3, 3AO, A2780 and SKOV3 was investigated. (**C**,**D**) Higher expression of GHET1 was significantly correlated with the tumor size and metastasis of ovarian cancer patients.

To investigate the function of GHET1 in ovarian cancer, the expression of GHET1 in ovarian cancer tissues and corresponding normal ovary was examined with the RT-qPCR analysis. Compared with the normal tissues, the expression of GHET1 was significantly enhanced in ovarian cancer tissues (Figure 1A). Furthermore, the expression of GHET1 was also detected in several ovarian cancer cell lines and normal ovarian epithelial cells. The result showed that the level of GHET1 was significantly increased in ovarian cancer cells (OVCAR3, SKOV3, 3AO and A2780) in comparison with that of the HOSEpiC (Figure 1B). These data suggested the up-regulation of GHET1 in ovarian cancers. To further characterize the aberrant expression of GHET1 in ovarian cancer, the correlation between the expression of GHET1 with the tumor size and metastasis stage of these patients was analyzed. As shown in Figure 1C,D, up-regulation of GHET1 was positively correlated with increased tumor size and distant metastasis of ovarian cancer. These results indicated that high expression of GHET1 was potentially involved in the progression of ovarian cancer.

### GHET1 modulated the proliferation of ovarian cancer cells

**Figure 2:**
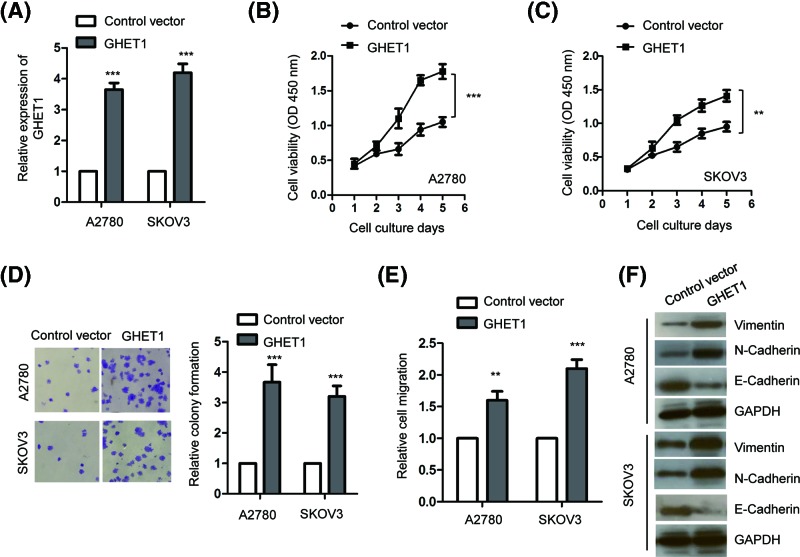
Overexpression of GHET1 promoted the proliferation of ovarian cancer cells (**A**) Relative expression of GHET1 was analyzed by RT-qPCR in both A2780 and SKOV3 cells after transfection of pcDNA-3.1 or pcDNA-3.1-GHET1. (**B**,**C**) The proliferation of ovarian cancer cells expressing control vector or GHET1 was measured with the MTT assay at the indicated times. (**D**) Crystal violet staining of colonies of both A2780 and SKOV3 cells transfected with control vector or GHET1. (**E**) The relative migration of ovarian cancer cells harboring control vector or GHET1 was detected. (**F**) The expression of vimentin, N-cadherin and E-cadherin in A2780 and SKVO3 cells transfected with GEHT1 or control vector was detected by Western blot. **P<0.01; ***P<0.001.

Since GHET1 was overexpressed in ovarian cancers, we next investigated the influence of GHET1 on the growth of ovarian cancer cells. To this end, A2780 and SKOV3 cells were transfected with the expression vector of GHET1 (pcDNA-3.1-GHET1) to enhance the expression of GHET1. As presented in Figure 2A, compared with the cells expressing empty vector pcDNA-3.1, the expression level of GHET1 was significantly higher in both SKOV3 and A2780 cells with the transfection of pcDNA-3.1-GHET1. The MTT assay showed that overexpression of GHET1 promoted the proliferation of both A2780 and SKOV3 cells (Figure 2B,C). To further confirm the growth-enhancing effect of GHET1, colony formation assay was performed with ovarian cancer cells expressing GHET1 or the control vector. The result suggested that overexpression of GHET1 promoted the colony formation of both A2780 and SKOV3 cells in comparison with the cell harboring control vector (Figure 2D). Additionally, the effect of GHET1 on the migration of ovarian cancer cells was also examined with the *in vitro* would-healing assay. The result showed that overexpression of GHET1 significantly promoted the migration capacity of both A2780 and SKOV3 cells (Figure 2E). To evaluate whether GHET1 affected the EMT of ovarian cancer cells, both A2780 and SKOV3 cells were transfected with GHET1 or the control vector, and the expression of EMT maker proteins including Vimentin, N-cadherin and E-cadherin were detected by western blot. The results showed that overexpression of GHET1 increased the level of vimentin and N-cadherin in both A2780 and SKOV3 cells (Figure 2F), while the level of E-cadherin was decreased with the transfection of GHET1. The result indicated the potential promotion effect of GHET1 on the EMT of ovarian cancer cells.

### Knockdown of GHET1 suppressed the proliferation and induced apoptosis of ovarian cancer cells

**Figure 3:**
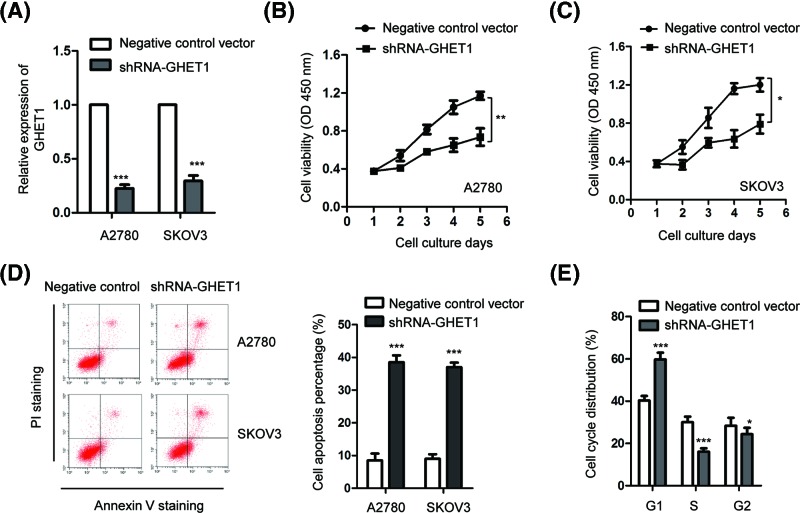
Knockdown of GHET1 suppressed the proliferation and induced apoptosis of ovarian cancer cells (**A**) Both A2780 and SKOV3 cells were transfected with negative control-shRNA or shRNA-GEHT1, and the knockdown efficiency was measured by the RT-qPCR assay. (**B**,**C**) The proliferation of ovarian cancer cells was detected by the MTT assay after transfection of negative control vector or shRNA-GHET1. (**D**) FACS assay was performed to compare the apoptosis rate of cell expressing control vector or depleted GHET1. (**E**) Down-regulation of GEHT1 in ovarian cancer cells induced G1 cell cycle arrest. *P<0.05; **P<0.01; ***P<0.001.

To further confirm the potential oncogenic function of GHET1 in ovarian cancer, the endogenous expression of GHET1 was down-regulated by transfecting shRNA-GHET1 into both A2780 and SKOV3 cells (Figure 3A). The influence of depleted GHET1 on the proliferation of ovarian cancer cells was detected by the MTT assay, which showed that down-regulation of GHET1 significantly suppressed the proliferation of ovarian cancer cells (Figure 3B,C). Considering the inhibitory effect of depleted GHET1 on the growth of ovarian cancer cells, we then examined the function of GHET1 in regulating the apoptosis of ovarian cancer cells. As shown in Figure 3D, the percentage of apoptotic cells was markedly increased with the depletion of GHET1 compared with the control group. Additionally, the cell cycle progression by flow cytometry demonstrated that knockdown of GHET1 significantly enhanced the distribution of OVCAR3 cells in the G0/G1 phase and deceased in the S phase (Figure 3E), which suggested the G1 cell cycle arrest with the down-regulation of GHET1. These results indicated that knockdown of GHET1 blocked the cell cycle progression and induced apoptosis of ovarian cancer cells.

### GHET1 up-regulated the expression of HIF1α via interacting with the E3 ubiquitin ligase VHL

**Figure 4:**
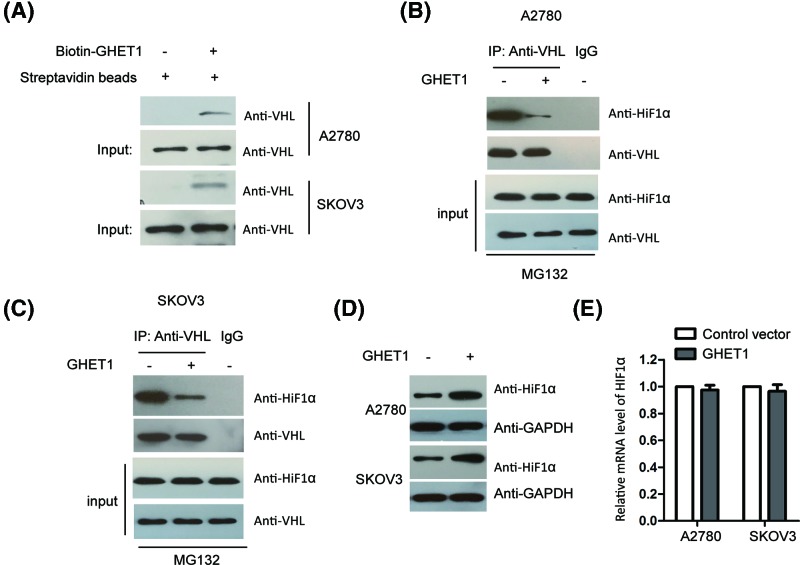
GHET1 interacted with VHL and up-regulated HIF1α (**A**) *In vitro* pull-down assay was performed to detect the binding between GHET1 and VHL. (**B**,**C**) Both A2780 and SKOV3 cells were transfected with or without GHET1. Co-immunoprecipitation assay was performed with anti-VHL antibody, and the interaction between VHL and HIF1α was detected with indicated antibody. (**D**) Ovarian cancer cells were transfected with control vector or GEHT1 and the expression of HIF1α was detected. (**E**) The mRNA level of GHET1 was investigated in both A2780 and SKOV3 cell expressing GEHT1.

To further characterize the underlying molecular mechanisms by which GHET1 modulated the growth of ovarian cancer cells, we screened the potential binding partners of GHET1 with the RNA pull-down and mass spectrometry analysis. The result showed that the E3 ubiquitin ligase VHL was a putative binding partner of GHET1 (Table 1). To confirm this, *in vitro* binding assay was performed by incubating biotin-labeled GHET1 with the lysates of A2780 and SKOV3 cells. As presented in Figure 4A, VHL was detected in the immunoprecipitation complex with GHET1, which suggested the interaction between GHET1 and VHL in ovarian cancer cells. As VHL was reported as the E3 ligase of HIF1α, we further investigated whether the binding between VHL and GHET1 affected the interaction between VHL and HIF1α. To this end, both SKOV3 and A2780 cells were transfected with or without pcDNA-3.1-GEHT1, and the interaction between VHL and HIF1α was checked with the co-immunoprecipitation assay. The data showed that overexpression of GHET1 decreased the binding between VHL and HIF1α in both SKOV3 and A2780 cells (Figure 4B,C). These results suggested that the interaction of GHET1 with VHL blocked its binding with HIF1α.

**Table 1:** The candidate binding partners of GHET1

Protein name	Molecular weight (KDa)	Full name
VHL	18	Von Hippel-Lindau
HnRNPA1	38	Heterogeneous nuclear ribonucleoprotein A1
RPL6	33	60s ribosomal protein L6
eIF4A	46	Eukaryotic initiation factor 4A
NPM	33	Nucleophosmin
PRMT5	73	Protein arginine N-methyltransferase 5
RPS7	22	40s ribosomal protein S7

To investigate whether the decreased binding between VHL and HIF1α affected the protein stability of HIF1α, A2780 and SKOV3 cells were transfected with GHET1 and the protein abundance of HIF1α was detected by the Western blot. The result showed that highly expressed GHET1 promoted the protein level of HIF1α (Figure 4D). The mRNA level of HIF1α was not significantly altered with the transfection of GHET1 (Figure 4E). Collectively, these results demonstrated that GHET1 up-regulated the expression of HIF1α via interrupting the interaction between VHL and HIF1α.

### GHET1 promoted the glycolysis of ovarian cancer cells

**Figure 5:**
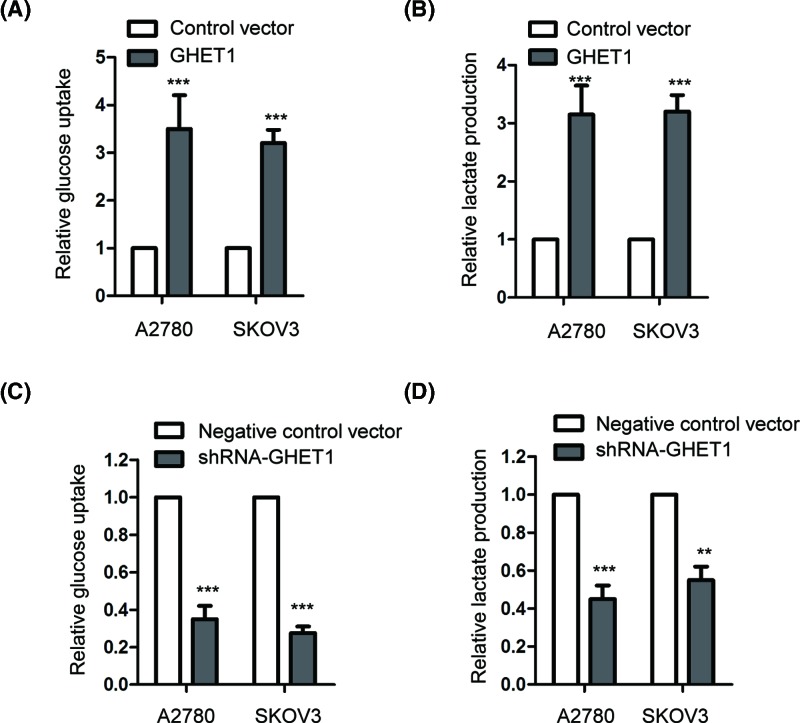
GEHT1 modulated the glycolysis of ovarian cancer cells (**A**) Both A2780 and SKOV3 cells were transfected with control vector or GEHT1. The glucose uptake and lactate production of the cells were measured. (**C**,**D**) The endogenous expression of GHET1 was down-regulated with the expression of shRNA-GEHT1. The glucose consumption and lactate production were detected. **P<0.01; ***P<0.001.

As GHET1 regulated the expression of HIF1α, we further detected the effect of GHET1 on the glycolysis of ovarian cancer cells. Both A2780 and SKOV3 cells were transfected with GHET1 or control vector, and then the glucose uptake as well as the lactate production of the cells were evaluated. As indicated in Figure 5A,B, overexpression of GHET1 significantly enhanced the glucose consumption and lactate production of both A2780 and SKOV3 cells, which indicated that overexpression of GHET1 promoted the glycolysis of ovarian cancer cells. To further support this conclusion, GHET1 was down-regulated by transfecting shRNA-GHET1 into the ovarian cancer cells and the glycolysis of the cells was examined. The results showed that compared with the control cells, depletion of GHET1 significantly suppressed the glucose consumption and lactate production in both A2780 and SKOV3 cells (Figure 5C,D). These data indicated that GHET1 was a novel regulator of the glycolysis in ovarian cancer cells.

## Discussion

The function of lncRNAs is involved in regulating a variety of biological processes including X-chromosome inactivation, genomic imprinting and RNA processing [20]. Recent years, the importance of lncRNAs in the initiation and progression of human disease, especially cancers, has drawn wide attention. Ovarian cancer is one of the most common malignancies among women worldwide. The high occurrence and mortality of ovarian cancer calls for the identification of novel factors that contribute to the progression of ovarian cancer. Although accumulating evidence has demonstrated the involvement of lncRNAs in human cancers, the function of lncRNAs in ovarian cancer remains largely unknown. In the present study, we characterized that GHET1 was highly expressed in ovarian cancer tissues and cell lines, which promoted the proliferation of ovarian cancer cells.

GHET1 was first identified in gastric cancer, which was highly expressed in cancer tissues and promoted the proliferation of gastric cancer cells via modulating expression of c-Myc [12]. The finding in bladder cancer suggested that GHET1 acted as an oncogene via promoting the EMT of the cells [13]. Consistent with these reports, our results found that GHET1 was overexpressed in ovarian cancers and correlated with the poor prognosis of cancer patients. Enhanced expression of GHET1 significantly promoted the proliferation and colony formation of ovarian cancer cells. These results demonstrated that overexpressed GEHT1 in ovarian cancer cells positively regulated the progression of cancers. *In vivo* studies that might support the potential oncogenic function of GHET1 in ovarian cancer are necessary in further investigations.

Aerobic glycolysis has been considered as the hallmark of cancers [14]. Targeting the glycolysis pathway to down-regulate the glucose metabolism of cancer cells is a novel strategy to suppress the progression of cancers. Accumulating evidence showed that lncRNAs modulated the glucose metabolism of cancer cells via diverse mechanisms [21,22]. In this study, our data uncovered that GHET1 contributed to the glycolysis of ovarian cancer cells. Mechanistically, GHET1 interacted with VHL, the E3 ubiquitin ligase of HIF1α, which blocked the binding between VHL and HIF1α in ovarian cancer cells. It is worth noticing that hypoxia or oncogenic events lead to the stabilization of HIF1α and trigger the transactivation of key enzymes in the glycolysis [23,24]. Inhibition of HIF1α has presented the capacity to block the tumorigenesis in animals via down-regulating the glycolysis. The protein stability of HIF1α was mainly regulated by the E3 ligase VHL-mediated polyubiquitination and 26S proteasome dependent degradation [25,26]. Therefore, modulating the interaction between VHL and HIF1α is critical to regulate the abundance of HIF1α. In the present study, we identified GHET1 as a novel binding partner of VHL, which consequently promoted the protein stability of HIF1α and enhanced the glucose metabolism of ovarian cancer cells. It would be interesting to investigate whether the modulation of GHET1 for the interaction of VHL and HIF1α also occurs in other types of cancers.

In conclusion, our results found that GHET1 was highly expressed in ovarian cancer tissues and correlated with the progressed condition of cancer patients. Overexpression of GHET1 promoted the glycolysis of ovarian cancer cells via modulating VHL-mediated degradation of HIF1α. These results uncovered the possible functional mechanism of GHET1 in ovarian cancer, which suggested that GEHT1 might be an attractive target to suppress the progression of ovarian cancer.

## Abbreviations

EMT: epithelial–mesenchymal transition
FBS: fetal bovine serum
FITC: fluorescein-5-isothiocyana
GHET1: gastric carcinoma high expressed transcript 1
HIF1α: hypoxia-inducible factor-1α
HOSEpiC: normal epithelial ovarian epithelial cells
lncRNAs: long non-coding RNAs
MTT: dimethyl thiazolyl diphenyl tetrazolium
PHD: proline hydroxylase domain
VHL: von Hippel-Lindau
